# 

**Published:** 1854-05

**Authors:** 


VOL. III. NEW SERIES. No. 5.

J. H. Hitchcock

### THE NORTH-WESTERN

### MEDICAL AND SURGICAL

### JOURNAL:

PUBLISHED MONTHLY.

AT TWO DOLLARS PER ANNUM IN ADVANCE.

EDITED BY

W. B. HERRICK, M.D.,

PROFESSOR OF ANATOMY AND PHYSIOLOGY IN RUSH MEDICAL COLLEGE; SURGEON TO THE U.S. MARINE HOSPITAL, ONE OF THE SURGEONS OF THE ILLINOIS GENERAL HOSPITAL, ETC.

AND

H. A. JOHNSON, A.M., M.D.

LECTURER ON PHYSIOLOGY AND HISTOLOGY IN RUSH MEDICAL COLLEGE.

---

MAY, 1854.

---

CHICAGO:

J. F. BALLANTYNE, PUBLISHER AND PRINTER,

NEW POST-OFFICE BUILDINGS, COR. OF CLARK AND RANDOLPH-STS., OPPOSITE THE SHERMAN HOUSE.

1854.

VOL XI. WHOLE SERIES. No. 5.

